# Potential Efficacy of Polyphenols and Isothiocyanates in the Management of Genitourinary Diseases: A Systematic Review of Preclinical and Clinical Studies

**DOI:** 10.3390/ijms27041660

**Published:** 2026-02-08

**Authors:** Eugenia Piragine, Pasquale Moretti, Salvatore Scarpulla, Vincenzo Calderone, Alma Martelli

**Affiliations:** 1 Department of Pharmacy, University of Pisa, Via Bonanno Pisano 6, 56126 Pisa, Italy; eugenia.piragine@unipi.it (E.P.); vincenzo.calderone@unipi.it (V.C.); 2 Interdepartmental Research Center “Nutrafood: Nutraceutica e Alimentazione per la Salute”, University of Pisa, Via del Borghetto 80, 56124 Pisa, Italy; 3 BIOENUTRA Srl, IV Traversa dx S.P. Bandiera, 74013 Ginosa, Italy; p.moretti@bioenutra.com (P.M.); s.scarpulla@bioenutra.com (S.S.)

**Keywords:** polyphenols, isothiocyanates, genitourinary diseases, preclinical studies, clinical studies

## Abstract

The global burden of genitourinary diseases is increasing worldwide, highlighting the need to identify new approaches for their prevention and treatment. In this context, polyphenols, isothiocyanates derived from plants of the *Brassicaceae* and *Moringaceae* botanical families, along with their natural sources, represent promising strategies, as they exhibit several health-promoting properties, including antioxidant, anti-inflammatory, and anti-fibrotic effects. However, a comprehensive overview of their benefits in the genitourinary system is currently unavailable. In this paper, we performed a systematic review of the literature by searching two scientific databases (MEDLINE and Scopus). A total of 27 preclinical studies and 16 clinical studies were included. Many studies have investigated the efficacy of isolated polyphenols in animal models of genitourinary diseases, as well as extracts or foods typically rich in polyphenols in humans, demonstrating potential benefits in the management of lithiasis, hyperoxaluria, prostatitis, benign prostatic hyperplasia, glomerulonephritis, vaginal dysbiosis, vaginosis, cystitis, and urinary tract infections. In contrast, few studies have examined isolated isothiocyanates, probably because their pharmacological role has only recently been recognized, and the results have been inconsistent. Further high-quality research is needed to confirm the preliminary evidence on polyphenols and to clarify the biological effects of isothiocyanates and their natural sources in this field.

## 1. Introduction

Genitourinary pathological conditions represent a growing global health threat, posing significant challenges not only for affected individuals, but also for caregivers and healthcare professionals [1,2,3]. In 2021, there were approximately 110 million prevalent cases of genitourinary diseases in Europe, 52 million in high-income North America, 28 million in Central Latin America, 6 million in Southern Latin America, 33 million in high-income Asia Pacific, and 4 million in Australasia [4]. In Europe, recent projections also suggest that the burden of genitourinary diseases will continue to rise over the next decade, especially among the elderly population [4]. This highlights the need to accelerate access to care, increase the perceived importance of these pathological conditions among healthcare professionals, and explore novel approaches for their management.

The most common noncancerous genitourinary diseases include, but are not limited to, urinary incontinence, cystitis, benign prostatic hyperplasia (BPH), urinary tract infections (UTIs), and lithiasis [3]. They not only have a significant impact on quality of life and social well-being [5,6,7] but, if left untreated or undertreated, they can increase the risk of developing various complications, including organ dysfunction [8,9] and genitourinary cancers [10,11,12]. Moreover, many of these diseases require recurrent antibiotic treatments, leading to a dramatic increase in developing antibiotic resistance [13,14]. In this scenario, the discovery of novel pharmacological and/or nutraceutical strategies is urgently needed.

In recent decades, particular attention has been given to many natural compounds with potential effects in the prevention and treatment of recurrent and chronic genitourinary pathological conditions. Among these, polyphenols, which can be found in plants [15] but also in microbial sources such as algae [16], have been extensively studied in both preclinical and clinical settings [17,18,19,20,21,22,23,24,25,26,27,28]. Based on their chemical structure, polyphenols can be categorized into two main groups (flavonoids and non-flavonoids), that can be further divided into several subgroups [29,30]. One of the most investigated is resveratrol, a stilbene primarily found in *Vitis vinifera* L. (grape) [31,32,33]. However, many other compounds have demonstrated promising health-promoting properties, including naringenin (from *Citrus* fruits, such as oranges and bergamot), quercetin (mainly from *Malus domestica* Borkh., apple; *Solanum licopersicum* L., tomato; and *Allium cepa* L., onion), and epigallocatechine-3-gallate (EGCG, from *Camellia sinensis* L., green tea) [34,35,36,37]. The growing interest in this class of natural compounds is not limited to the prevention and treatment of genitourinary diseases but also extends to cardiovascular diseases [17,18,19], neurological disorders [20,21,22], cardiometabolic diseases [23,24,25], type 2 diabetes [26], and cancer [27,28]. It is important to note that foods and extracts rich in polyphenols exhibit the same beneficial properties as isolated compounds [38,39,40,41], suggesting their key role in promoting biological effects.

Another class of particular interest is isothiocyanates (ITCs), which are derived from the hydrolysis of the corresponding glucosinolates (GLs), a reaction catalyzed by the plant enzyme myrosinase and, to a lesser extent, by sulfur-reducing bacteria in the human gut [30,42]. ITCs exert beneficial properties in multiple systems, including the cardiovascular system [43,44,45,46] and the central nervous system [47,48]. These effects may be at least partly attributed to the ability of natural ITCs to slowly release hydrogen sulfide (H_2_S), a gasotransmitter with health-promoting properties [49,50,51], whose biosynthesis is reduced during aging or under disease conditions [52]. Notably, vegetables and plant extracts particularly rich in GLs, such as *Brassica oleracea* L. (broccoli) and *Eruca sativa* Mill. (rocket salad or arugula), have also shown beneficial effects in both preclinical and clinical studies across a range of pathological conditions, including hypertension, type 2 diabetes, cardiovascular and metabolic diseases, gastrointestinal disorders, and cancer [43,53,54,55,56,57,58,59,60,61,62,63,64].

**Figure 1:**
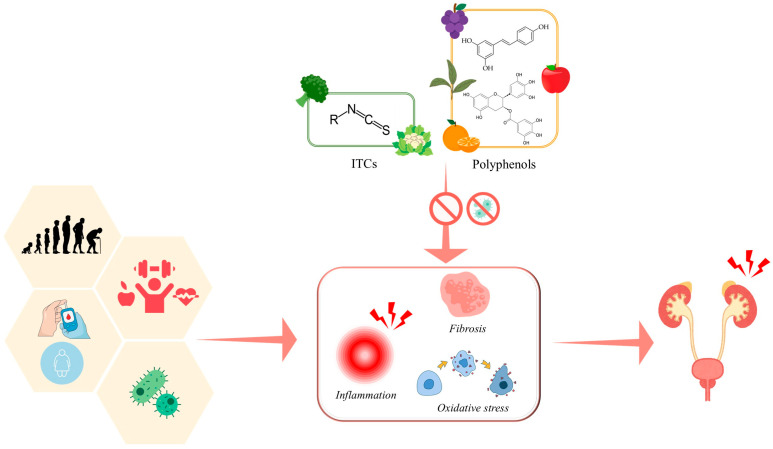
Schematic representation of the hypothesized mechanisms by which isothiocyanates (ITCs) and polyphenols could prevent and manage genitourinary diseases. From left to right: aging, lifestyle factors, metabolic disorders, and microbial infections may contribute to the development of genitourinary pathological conditions. ITCs and polyphenols could modulate key pathogenic processes by exerting antimicrobial, anti-fibrotic, anti-inflammatory and antioxidant effects. The horizontal red arrows represent the consequences of exposure to risk factors associated with genitourinary diseases, while the vertical red arrow with prohibition symbols indicates the inhibitory effects of polyphenols and ITCs.

To date, the effects of polyphenols and ITCs in the prevention and treatment of genitourinary diseases remain poorly investigated. The potential pharmacological role of these natural compounds is attributed to their antioxidant, anti-inflammatory, anti-fibrotic, and antimicrobial properties [65,66,67,68]. These mechanisms could modulate key pathogenic processes associated with aging, metabolic disorders, chronic inflammation, lifestyle factors, and microbial infections, all of which may contribute to the development of genitourinary pathological conditions [69,70,71,72,73] (Figure 1). However, the available studies on polyphenols and ITCs in this field are highly heterogeneous in terms of the animal model used and the patient population examined. The aim of this systematic review is to summarize current knowledge and identify areas of research that remain unexplored. While a relatively large number of preclinical studies have focused on the potential efficacy of isolated compounds in animal models of genitourinary pathologies, very few clinical trials have investigated their effects on genitourinary health in humans. Therefore, to provide a comprehensive overview of the existing evidence, clinical studies focusing on extracts or foods typically rich in polyphenols or GLs were also examined.

## 2. Methods

This systematic review followed the PRISMA guidelines. No protocol was prepared, and the review was not registered in a public database.

### 2.1. Search Strategy

MEDLINE (via Pubmed) and Scopus databases were searched for articles published up to 15 July 2025. The search strategy (Supplementary Materials S1) included a term related to the compounds of interest and another related to the pathological condition under study. These two terms were combined using the Boolean operator “AND”.

### 2.2. Inclusion Criteria

Preclinical in vivo studies evaluating the efficacy of sub-chronic or chronic treatments (lasting at least one week) with isolated polyphenols or ITCs in rodent models of genitourinary diseases were included in the systematic review. Rodent models (mice and rats) were selected because of their high anatomical similarity to humans [74,75]. Priority was given to in vivo studies due to their greater potential for translation into clinical practice compared to in vitro studies, which lack physiological complexity and do not take biokinetics into account [76].

Clinical studies were included if they assessed the efficacy of isolated polyphenols and/or ITCs, or of extracts or foods typically rich in polyphenols or GLs, in patients with genitourinary diseases or in healthy individuals, provided that the results were related to genitourinary health.

### 2.3. Exclusion Criteria

Reviews (both narrative and systematic), meta-analyses, studies reporting previously published data, abstracts, editorials, letters to the editor, case reports, and articles published in languages other than English were excluded.

### 2.4. Selection of Studies

Rayyan, a web-based screening tool [77], was used to perform the systematic review. Duplicates were removed, and titles and abstracts were screened to categorize records as irrelevant or potentially eligible. The full texts of the potentially eligible studies were retrieved or requested directly from the corresponding author, if unavailable. The entire process was conducted independently by two authors (E.P. and A.M.). Any disagreements were discussed and resolved with a third author (V.C.).

**Figure 2:**
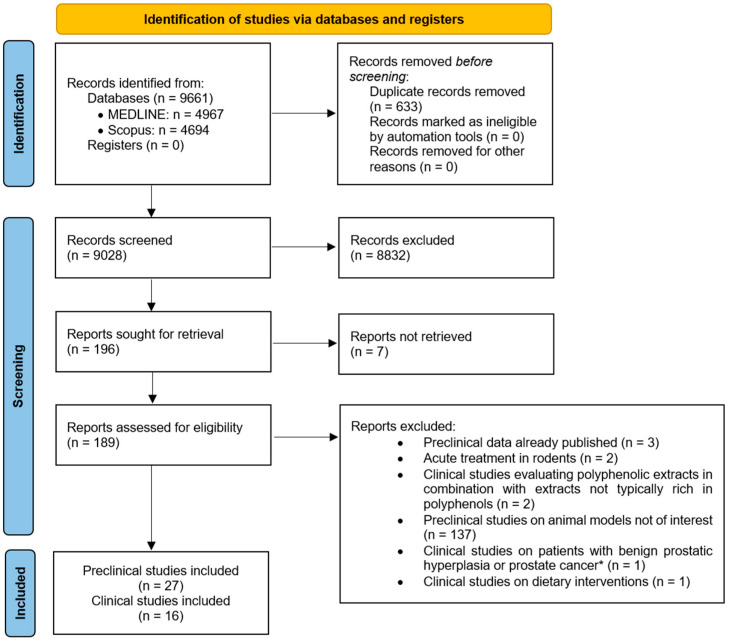
PRISMA flow diagram. Symbol legend: * no distinction between patient populations.

The study selection process is shown in Figure 2. A total of 4967 and 4694 records were identified from MEDLINE and Scopus, respectively. After the removal of duplicates, 9028 titles and abstracts were screened. Of these, 8832 records were excluded and 7 potentially eligible full texts were requested from the corresponding authors but could not be retrieved. Therefore, 189 articles were assessed for eligibility. Among these, 146 records were excluded for the following reasons: (i) preclinical data already published (N = 2); (ii) evaluation of acute treatments in rodents (N = 2); (iii) assessment of polyphenolic extracts in combination with extracts not typically rich in polyphenols (N = 2); (iv) preclinical studies on animal models not of interest (N = 137); (v) clinical studies on patients with BPH or cancer patients, without distinction between patient populations (N = 1); or (vi) clinical studies on dietary interventions with non-standardized portion sizes (N = 1). At the end of the screening process, 27 preclinical studies and 16 clinical studies were included in the systematic review.

### 2.5. Data Extraction

Data were extracted independently by two authors (E.P. and A.M.) using a standardized Microsoft Excel (version 2025) spreadsheet, which included predefined fields for each variable of interest. For preclinical studies, the extracted data included: (a) rodent model; (b) compound; (c) daily dosage and administration route; (d) number of animals; (e) length of treatment; (f) main results (efficacy and inefficacy); and (g) histological examination findings. For clinical studies, extracted information included: (a) study design; (b) population characteristics; (c) number of patients, age, and percentage of men; (d) compound/extract/food; (e) daily dosage, administration route, dosage form, and follow-up duration; and (f) main results. The primary outcome was genitourinary health (e.g., changes in functional parameters or in the expression of specific biological markers). Additional data on systemic oxidative stress and inflammatory markers were also extracted. Results were reported descriptively, with no formal synthesis, data transformations, or imputations performed. Missing or unclear data were not assumed or estimated, and all data were reported as presented in the original studies. Any discrepancies between the two authors were resolved by discussion and, when necessary, consultation with a third author (V.C.).

## 3. Results and Discussion

### 3.1. Preclinical Studies

**Figure 3:**
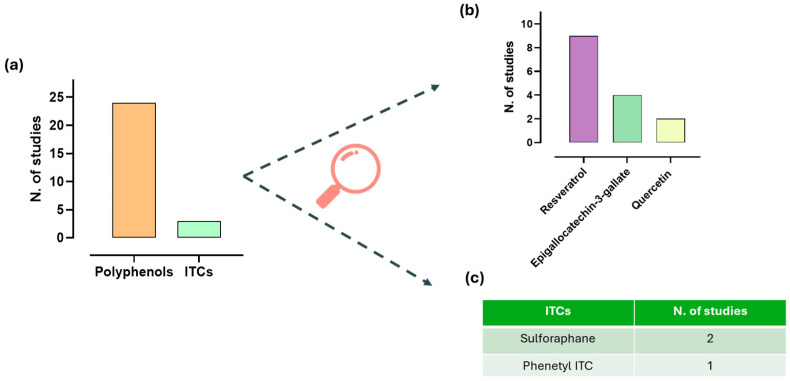
Isolated compounds investigated in preclinical studies included in the systematic review. The histograms show the number of studies on isothiocyanates (ITCs) and polyphenols (**a**) and the number of studies on the most frequently investigated polyphenols (i.e., those evaluated in more than one study) (**b**). The table shows the number of studies focusing on specific ITCs (**c**).

Out of the 27 preclinical studies included, 24 focused on polyphenols [78,79,80,81,82,83,84,85,86,87,88,89,90,91,92,93,94,95,96,97,98,99,100,101], while three evaluated the potential benefits of ITCs in rodent models of genitourinary pathological conditions [102,103,104] (Figure 3). Among polyphenols, the most investigated compound was resveratrol [79,83,84,85,91,93,94,98,99], followed by EGCG [89,92,97,101] and quercetin [82,89]. As concerns ITCs, sulforaphane was examined in two studies [103,104], while phenethyl ITC was assessed in only one article [102].

**Figure 4:**
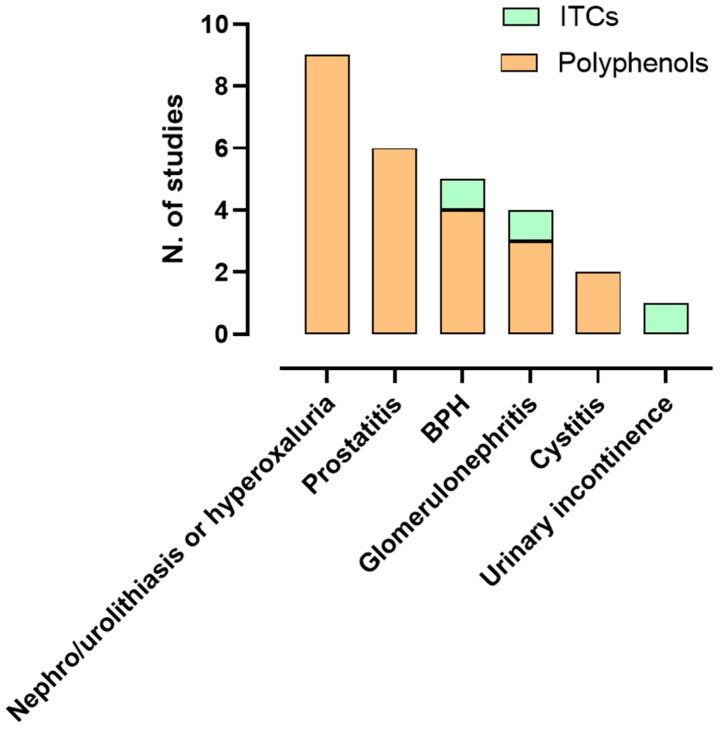
Rodent models of genitourinary pathological conditions used in preclinical studies included in the systematic review. The histograms show the number of studies on polyphenols (orange bars) and isothiocyanates (ITCs; green bars), stratified by rodent models of genitourinary diseases. Other abbreviations: BPH, benign prostatic hyperplasia.

Figure 4 shows the number of studies categorized by rodent models of genitourinary pathological conditions. Studies on polyphenols focused on nephrolithiasis, urolithiasis, or hyperoxaluria [80,81,82,84,88,89,90,91,96], followed by prostatitis [83,86,93,94,98,99], BPH [78,79,95,101], glomerulonephritis [85,92,97], and cystitis [87,100]. The three studies on ITCs focused on BPH [102], glomerulonephritis [103], and urinary incontinence [104].

The main characteristics and findings of the preclinical studies included in the systematic review are reported in Table 1 and Table S1 and summarized in the following sections.

#### 3.1.1. Polyphenols

- Nephro/urolithiasis and hyperoxaluria

Nine studies investigated the potential efficacy of isolated polyphenols in rodent models of nephro/urolithiasis or hyperoxaluria. As concerns experimentally induced nephrolithiasis, a 24-day oral treatment with epicatechin administered in drinking water (200 mg/L) significantly reduced renal calcium content, increased creatinine urinary levels, and decreased urinary calcium, phosphorus, and pH. However, no significant effects were observed on diuresis, crystal deposition, urinary oxalate and magnesium levels, or renal phosphorus and magnesium content. These findings suggest that while epicatechin may alleviate kidney injury, it does not directly affect crystal accumulation or promote their elimination [81].

A 5-week oral treatment with quercetin (10 mg/kg/day) significantly reduced urinary oxalate (but not calcium), plasma urea, plasma oxalate, and circulating malondialdehyde (MDA) levels, a marker of oxidative stress. Histological analysis revealed that quercetin induced a mild reduction in glomerular vessels congestion, mononuclear cell infiltration, and narrowing of tubular lumens, particularly in the proximal tubules, although no statistical analysis was performed [82]. In another study, a 13-day oral treatment with quercetin (10 mg/kg/day), EGCG (150 mg/kg/day), or their combination significantly reduced urea, creatinine, calcium, uric acid, phosphorus, potassium, and chloride levels in both urine and serum of rats with nephrolithiasis. In addition, polyphenols decreased catalase (CAT) activity and lipid peroxidation (LPO) in the kidney. Histological analysis showed that quercetin and EGCG restored kidney histoarchitecture by reducing crystals deposits and tubular dilation, although no statistical analysis was performed [89]. An 8-week oral treatment with caffeic acid (20 and 40 mg/kg/day) at the highest dose significantly increased urinary volume in rats with experimentally induced nephrolithiasis. In addition, both doses enhanced urinary citrate excretion and reduced oxalate excretion, thereby potentially reducing the risk of stone formation. Histological analysis revealed that treatment with caffeic acid restored the normal renal architecture and cleared crystal deposits from the renal tubules, although no statistical analysis was reported for these findings. Notably, these effects were observed whether caffeic acid was administered concurrently with the nephrolithiasis inducer glycol from day 1 or initiated from week 4 [96]. A 5-week oral treatment with resveratrol (10 mg/kg/day) led to a significant decrease in urinary and plasma oxalate, plasma urea, and circulating MDA levels. Histological analysis showed a reduction in renal tissue alterations (i.e., congestion of glomerular vessels in the cortex and medulla, mononuclear cell infiltration, and narrowing of the tubular lumen, particularly in the proximal tubules), although no statistical analysis was performed. No significant differences were observed in serum or urinary calcium levels [91]. Finally, a 6-day intraperitoneal treatment with combinations of citric acid and either gallic acid, ellagic acid, protocatechuic acid, or pyrogallic acid in rats with nephrolithiasis markedly reduced renal calcium oxalate (CaOx) deposition and renal injury score, as well as blood urea and serum creatinine levels. However, no statistical analysis was carried out [88]. Overall, these results suggest the potential benefits of quercetin, EGCG, resveratrol, caffeic acid, and hydroxybenzoic acid derivatives (such as gallic acid, ellagic acid, protocatechuic acid, or pyrogallic acid) in alleviating kidney injury and reducing stone formation in rodent models of nephrolithiasis. Furthermore, quercetin, resveratrol and EGCG may also mitigate oxidative stress. However, future studies using rigorous statistical analyses are needed to confirm these findings.

Only one study has examined the effects of polyphenols in a rodent model of urolithiasis, demonstrating that a 28-day oral treatment with rutin (20 mg/kg/day) or curcumin (60 mg/kg/day) significantly lowered urinary and renal CaOx levels, as well as serum creatinine, LPO, and the number of CaOx deposits in the kidneys. Histological analysis revealed that both polyphenols reduced kidney damage and interstitial fibrosis with dense eosinophilic infiltration, although no statistical analysis was performed. Regarding potentially undesirable effects, curcumin, but not rutin, led to a reduction in urine volume [80].

As concerns hyperoxaluria, a 21-day oral treatment with resveratrol (5 and 10 mg/kg/day) significantly reduced the number of urinary CaOx crystals and induced a marked, dose-dependent increase in the renal expression of the antioxidant enzymes glutathione peroxidase (GPx), CAT, and superoxide dismutase (SOD). In addition, resveratrol significantly lowered circulating MDA levels [84]. Similarly, a 4-week oral treatment with an unspecified green tea polyphenol (10, 30, and 300 mg/kg/day) significantly reduced the ethylene glycol-induced upregulation of renal protein expression of the antioxidant transcription factor nuclear factor erythroid 2-related factor 2 (Nrf2), as well as its downstream targets heme oxygenase-1 (HO-1) and NAD(P)H: quinone oxidoreductase 1 (NQO1), but only at the highest dose. Histological analysis showed a dose-dependent decrease in kidney swelling and inhibition of stone formation. The highest dose restored the basic shape of the kidney and renal tissue architecture, with glomeruli appearing normal and intact, although statistical analysis was not performed [90]. Therefore, resveratrol and green tea polyphenols appear to be effective in counteracting oxidative stress and reducing stone formation in rodent models of hyperoxaluria, but further high-quality studies are required to confirm these findings.

- Prostatitis

Six studies examined the effects of isolated polyphenols in various rodent models of prostatitis. A 10-day oral treatment with resveratrol (10 mg/kg/day) significantly reduced fibrosis, inflammatory cell infiltration [83,93,99], and the levels of the pro-inflammatory markers interleukin (IL)-6, IL-18, and tumor necrosis factor-α (TNF-α) in prostate tissue [93]. In addition, resveratrol improved overactive bladder symptoms by reducing bladder maximum capacity, residual urine volume, and maximum voiding pressure [83,98,99]. Histological analysis revealed a reduction in prostatic epithelial height, reorganization of the bladder detrusor muscle layer, as well as a decrease in diffuse inflammation, collagen accumulation, and glandular lumen diameter in the prostates of treated rats, although no statistical analysis was performed [83,99]. A higher dose of resveratrol (20 mg/kg/day for 10 days, orally) also reduced inflammatory cell infiltration, fibrosis and the levels of pro-inflammatory markers (i.e., IL-1β and TNF-α) in the prostate, probably by decreasing the expression of the pro-inflammatory transcription factor nuclear factor kappa-light-chain-enhancer of activated B cells (NF-kB) and increasing that of the anti-inflammatory and antioxidant enzyme sirtuin 1 (SIRT1) [94]. Finally, a 4-week oral treatment with oligonol (60 mg/kg/day) significantly increased the activity of the antioxidant enzyme GPx in both plasma and prostatic tissue of rats with prostatitis. Histological analysis revealed that oligonol reduced the presence of inflammatory cells in the glandular lumina, decreased the severity of inflammation, and improved the morphology of epithelial and stromal cells [86]. These findings suggest that resveratrol and oligonol may counteract inflammation and potentially prevent histological alterations in rodent models of prostatitis. However, future studies using rigorous statistical analyses are needed.

- BPH

Four studies demonstrated that sub-chronic and chronic treatment with isolated polyphenols is effective in rodent models of BPH. A 2-week oral treatment with resveratrol (100 mg/kg/day) significantly reduced prostate weight, hyperplasia, prostate contractility ex vivo, and reactive oxygen species (ROS) production [79]. A 28-day oral treatment with protocatechuic acid (40 mg/kg/day) significantly decreased prostate weight and reduced oxidative stress and inflammatory markers in prostatic tissue, including myeloperoxidase (MPO) activity, LPO, and MDA and nitric oxide (NO) levels. In addition, it lowered systemic inflammatory markers in the blood, such as MPO activity and levels of NO, IL-1β and TNF-α, suggesting both localized and systemic anti-inflammatory effect. The activity of the antioxidant enzyme SOD was significantly increased, whereas that of glutathione-S-transferase (GST) remained unchanged in the prostate tissue at the end of treatment. Histological examination, although not statistically analyzed, indicated a reduction in prostatic hyperplasia, with no significant effects on other histopathological features in the BPH model [78]. A 4-week oral treatment with EGCG (50 and 100 mg/kg/day) significantly decreased androgen and estrogen receptor expression in prostatic tissue and exhibited anti-inflammatory and antioxidant effects. Specifically, EGCG: (i) reduced prostatic MDA levels and increased the activities of SOD, CAT and GPx, as well as levels of glutathione (GSH) and total thiols; (ii) lowered ILs and TNF-α levels, and cycloxigenase-2 (COX-2) activity; (iii) ameliorated prostatic epithelial–mesenchymal transition and reduced markers of angiogenesis and fibrosis, including vascular endothelial growth factor (VEGF) and transforming growth factor-β (TGF-β), respectively. Histological examination showed a reduction in prostatic morphological changes (i.e., epithelial cells expansion, papillary protuberances, and collagen deposition), although no statistical analysis was performed [101]. Finally, a 28-day oral treatment with diosmin (20 and 40 mg/kg/day) dose-dependently reduced LPO and xanthine oxidase (XO) activity, as well as MDA and uric acid levels in prostatic tissue. In addition, treatment with diosmin led to an increase in prostatic levels of CAT, GST, GPx, glutathione reductase (GR), and GSH, accompanied by a significant decrease in serum prostate-specific antigen (PSA) levels. Histological analysis showed a reduction in epithelial thickening and lumen area, as well as an increase in the number of papillary fronds. However, statistical analysis was not performed, and future studies are required to confirm these preliminary findings [95].

- Glomerulonephritis

Three studies investigated the effects of polyphenols in rodent models of glomerulonephritis. Two of these focused specifically on EGCG. In one study, a 3-week oral treatment (25 and 50 mg/kg/day) significantly reduced serum creatinine, proteinuria and mortality rate. In addition, EGCG ameliorated renal injury, as evidenced by improvements in glomerular damage, crescent formation, and tubulointerstitial injury. EGCG also reduced renal infiltration of inflammatory cells (i.e., macrophages and lymphocytes) and exhibited antioxidant effects by increasing renal GSH levels, reducing both renal and urinary MDA levels, and enhancing the expression of the antioxidant proteins Nrf2, glutamate-cysteine ligase catalytic subunit (GCLC), glutamate-cysteine ligase modifier subunit (GCLM), SIRT1 and GPx, but not HO-1, in the kidney [97]. In another study, a 15-day oral treatment with EGCG (50 mg/kg/day) significantly reduced proteinuria, serum creatinine levels, histopathological alterations, and renal injury, as indicated by improvements in the glomerulonephritis score, crescent formation, and tubulointerstitial injury score. EGCG also decreased macrophage and lymphocyte infiltration in the kidney. The anti-inflammatory effect was partly mediated by reduced expression of MPO and inducible nitric oxide synthase (iNOS) in the kidney, as well as by lower levels of total NO metabolites in both kidney and urine. Finally, EGCG attenuated oxidative stress by reducing MDA and hydrogen peroxide (H_2_O_2_) levels and by enhancing renal GPx expression and CAT activity. However, no significant effects were observed on nitrosative stress in kidney tissue. Notably, EGCG also reduced proteinuria, serum creatinine levels, and mortality when treatment was initiated on day 7 following the induction of glomerulonephritis [92].

One study focused on the effects of a 3-week oral treatment with resveratrol or piperine (40 mg/kg/day) in mice with glomerulonephritis. Both polyphenols significantly reduced albuminuria and serum creatinine levels, and urinary uric acid excretion. In addition, they promoted antioxidant effects by increasing renal GSH levels and SOD activity (both compounds), as well as CAT activity (piperine only), and by reducing thiobarbituric acid reactive substances (TBARS). Finally, resveratrol and piperine decreased serum uric acid and blood urea nitrogen levels, further supporting their potential role in ameliorating kidney damage under glomerulonephritis conditions [85].

- Cystitis

Only two studies focused on the potential efficacy of polyphenols in rodent models of cystitis. A 3-day oral treatment with rosmarinic acid (50 mg/kg/day) significantly increased the micturition interval and reduced urothelial thickness, prostaglandin E2 release, levels of inflammatory markers (i.e., IL-6), and MPO activity in the bladder. However, no significant changes were observed in micturition volume or bladder weight [87]. In another study, a one-week oral treatment with pterostilbene (10, 20 and 40 mg/kg/day) dose-dependently lowered bladder bleeding, histopathological scores, edema, pain, and inflammation (as indicated by decreased levels of IL-1β, IL-18, and NOD-like receptor family, pyrin domain containing 3–NLRP3—inflammasome components in bladder tissue). These anti-inflammatory effects were supported by histological evidence of reduced inflammatory cell infiltration. Pterostilbene also decreased bladder weight and the bladder weight-to-body weight ratio. Finally, it attenuated oxidative stress by lowering MDA levels and upregulating the expression of antioxidant proteins HO-1, SOD, and Nrf2 in bladder tissue [100]. Taken together, these preliminary findings indicate that polyphenols may exert beneficial effects in rodent models of cystitis, in part by reducing oxidative stress and inflammation.

#### 3.1.2. ITCs

One study examined the effects of a 3-week oral treatment with phenethyl ITC (12 µmol/rat/day) in a rodent model of BPH. The isolated ITC reduced prostate weight and hyperplasia and showed a trend toward decreased androgen receptor expression and testosterone-mediated cell cycle progression. However, histological examination revealed persistent increase in the nucleus-to-cytoplasm ratio, along with the presence of vesicular nuclei, prominent nucleoli, and vacuolated cytoplasm in the prostatic tissue of phenethyl ITC-treated rats [102].

Two preclinical studies evaluated the potential beneficial effects of sulforaphane in rodent models of other genitourinary diseases. One study reported that a 16-week intraperitoneal treatment with sulforaphane (1 mg/kg/day) significantly reduced the urinary albumin/creatinine ratio, glomerular volume, and kidney damage in a mouse model of glomerulonephritis [103]. The other study demonstrated that a 30-day intraperitoneal treatment with sulforaphane (0.5 mg/kg/day) significantly increased peak voiding pressure and micturition interval, lowered MDA levels, and enhanced the activity of GPx and SOD in the urethra of female rats with urinary incontinence. In addition, sulforaphane reduced cell apoptosis in the urethral sphincter and normalized urethral sphincter muscle histology and collagen content, although no statistical analysis was performed [104]. Due to the limited number of studies on ITCs and the absence of statistical analysis for some outcomes, further research is warranted to confirm these preliminary findings and to better elucidate the potential role of ITCs in the treatment of genitourinary pathological conditions.

#### 3.1.3. Summary

Currently available preclinical evidence on the beneficial effects of isolated polyphenols and ITCs in the treatment of genitourinary pathological conditions remains limited. Although these natural compounds have demonstrated promising anti-inflammatory and antioxidant properties that could help counteract local and systemic damage, histological analyses—which are essential for evaluating the effects on tissues and drawing definitive conclusions—are often not accompanied by statistical analyses.

In summary, the preliminary findings of the studies included in this systematic review suggest potential effects of: (i) quercetin, EGCG, caffeic acid, and resveratrol in ameliorating kidney injury in rodent models of nephrolithiasis; (ii) rutin and curcumin in reducing CaOx levels under urolithiasis conditions; (iii) resveratrol and green tea polyphenols in alleviating hyperoxaluria; (iv) oligonol and resveratrol in preventing histological alterations in rodent models of prostatitis; (v) resveratrol, protocatechuic acid, diosmin, and EGCG in reducing inflammation, oxidative stress, and prostate weight in experimentally induced BPH; (vi) EGCG, resveratrol, and piperine in alleviating glomerulonephritis; and (vii) rosmarinic acid and pterostilbene in counteracting oxidative stress and inflammation, as well as in reducing bladder weight and/or urothelial thickness in rodent models of cystitis (Table 1 and Table S1).

Very few preclinical studies have focused on isolated ITCs, demonstrating that phenethyl ITC produced inconclusive effects in rodent models of BPH, while sulforaphane potentially alleviated glomerulonephritis and urinary incontinence in mice and rats. However, further high-quality studies are needed to confirm these preliminary findings.

### 3.2. Clinical Studies

**Figure 5:**
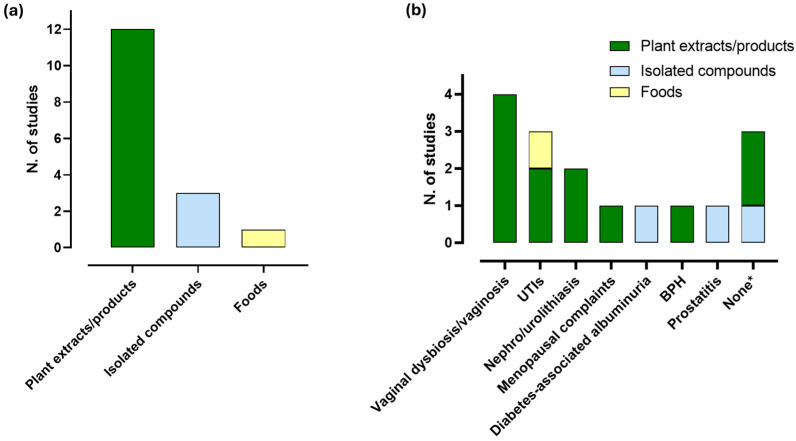
Summary of the clinical studies included in the systematic review. The histograms show the number of studies investigating plant extracts/products, isolated compounds, or foods (**a**) and the number of studies on plant extracts/products (green bars), isolated compounds (light blue bars) and foods (yellow bar), stratified by genitourinary diseases (**b**). Symbol legend: * assessment of genitourinary outcomes in healthy individuals or in people with metabolic syndrome. Abbreviations: BPH, benign prostatic hyperplasia; UTIs, urinary tract infections.

No study has investigated the effects of ITCs or their natural sources in patients with genitourinary diseases. In contrast, 12 clinical studies have examined isolated polyphenols and polyphenol-rich extracts or products. These include cranberry juice [105,106,107], cranberry extract (derived from the whole fruit [106], leaves [107], or unspecified plant parts [108]), whole tomato extract [109], fermented soy (from *Glycine max* L. seeds) combined with resveratrol [110], *Cymbopogon olivieri* extract (derived from the whole plant) [111], wax propolis extract [112], a mixture of extracts from *Castanea sativa* Mill., *Serenoa repens* (W. Bartram) Small, and *Vaccinium macrocarpon* Ait. [113], green tea bags [114], pomegranate extract (derived from the whole fruit) [115], and a mixture of Tea Tree Oil, *Tabebuia avellanedae* cortex extract, *Juglans regia* L. leaf extract, and hydroxytyrosol [116]. In addition, three studies investigated the potential efficacy of supplementation with isolated compounds (green tea polyphenols [117] and resveratrol [118,119]), while only one study assessed the effects of a food product (i.e., sweetened dried cranberries) [120] (Figure 5a).

As concerns the genitourinary diseases investigated, studies on plant extracts or products focused on vaginal dysbiosis/vaginosis [105,111,112,116], UTIs [108,113], nephrolithiasis or urolithiasis [114,115], menopausal complaints [110], and BPH [109]. Studies on isolated compounds assessed diabetes-associated albuminuria [117] and prostatitis [119], while the only one study on foods examined the effects of sweetened dried cranberries in patients with UTIs [120]. Finally, three studies did not include patients with genitourinary pathologies but evaluated genitourinary outcomes in healthy individuals [106,107] or in people with metabolic syndrome [118] (Figure 5b).

The main findings of the clinical studies included in the systematic review are shown in Table 1 and Table S2 and summarized in the following sections.

#### 3.2.1. Vaginal Dysbiosis/Vaginosis

A total of four studies investigated the effects of polyphenol extracts or products in patients with vaginal dysbiosis/vaginosis. One study was a double-blind, randomized, placebo-controlled, cross-over trial that focused on a 15-day supplementation with cranberry juice (8 fl oz/day, standardized in polyphenols) in postmenopausal women. Cranberry juice had minimal effect on the vaginal microbiota in women with *Lactobacillus*-dominated microbiota, but preserved key bacteria (i.e., *Firmicutes* and *Actinobacteria*) in women with more diverse microbiota. In women with dysbiosis, cranberry juice replaced the highly abundant *Streptococcus* with *Firmicutes* and *Actinobacteria*, suggesting a positive effect on the restoration of the microbiota [105]. In a double-blind randomized controlled trial (RCT), topical treatment of women with bacterial vaginosis using vaginal capsules containing *Cymbopogon olivieri* extract (500 mg/day for one week, standardized in polyphenols, derived from the whole plant) markedly reduced the percentage of patients with vaginal burning, itching, malodor, abundant discharge, and pH > 4.5. Moreover, *Cymbopogon olivieri* extract lowered the positive whiff test and the number of clue cells, indicating beneficial effects against bacterial vaginosis. Notably, these effects were comparable to those of the reference compound, the antibiotic metronidazole (control) [111]. In another RCT, topical treatment of women with vaginal candidiasis using wax propolis (5.0%) from *Tetragonula* sp. (one vaginal ovule/day for one week) inhibited the growth of *Candida albicans*, with no significant difference between the intervention group and the control group (the antifungal nystatin). Importantly, the number of dropouts due to infections was comparable between groups. However, the masking procedure was unknown and the wax propolis was not standardized in polyphenols, which introduced a potential risk of bias [112]. Finally, a 3-month oral supplementation with Micotirosolo^®^ (a commercial product particularly rich in hydroxytyrosol, but also containing Tea Tree Oil, *Tabebuia avellanedae* cortex extract, and *Juglans regia* L. leaf extract) in women with recurrent vulvovaginal candidiasis reduced clinical symptoms and vaginal signs, including pruritus, burning, itching, dryness, vulvar erythema, vaginal discharge, tenesmus, and dyspareunia. In addition, Micotirosolo^®^ enhanced the patient’s impression of global improvement, with 85% of patients reporting being “very much better”. However, the study was a single-arm clinical trial, and the daily dose of the commercial polyphenol-rich product was unknown. This could affect the overall methodological quality of the study [116].

#### 3.2.2. UTIs

Three clinical trials examined the effects of polyphenol extracts or polyphenol-containing foods in children and adult patients with UTIs. One was a controlled clinical study focused on a mixture of *Castanea sativa* Mill., *Serenoa repens* (W. Bartram) Small, and *Vaccinium macrocarpon* Ait. extracts, standardized in polyphenols, in nephropathic patients with recurrent UTIs. The intervention group received one capsule/day (6.21 mg of polyphenols/day) for 6 weeks, while the control group was not treated. Oral supplementation with the extract mixture reduced erythrocyte sedimentation rate in males and leukocytes in the urinary sediment in both males and females. The intervention also significantly reduced urinary bacterial flora in males and prevented recurrence of UTIs in the entire population. In terms of systemic effects, the polyphenol-containing extract reduced free oxygen radical levels and improved defense against oxygen free radicals in males, but not in females [113]. In a double-blind RCT, oral supplementation with cranberry extract syrup (Urell^®^, ~5.6 mg/kg/day of cranberry extract, standardized in polyphenols) in children aged 1 month to 13 years with a history of recurrent UTIs, followed for up to 1 year, resulted in an UTI rate similar to that observed in the control group receiving the antibiotic trimethoprim (35.0% vs. 28.0% in children under 1 year, and 26.0% vs. 35.0% in children over 1 year, respectively). In addition, children receiving Urell^®^ showed a percentage of multidrug-resistant bacteria in urine cultures comparable to that of the control group (22.9% vs. 33.3%). In both groups, recurrent UTIs were mainly caused by *Escherichia coli*. Notably, cranberry syrup administration was associated with high urinary levels of hydroxycinnamic and hydroxybenzoic acids [108], which have been shown to inhibit bacterial biofilm formation and reduce surface hydrophobicity in previous studies [121,122]. Finally, daily consumption of sweetened dried cranberries (42 g/day) for 2 weeks by women with a history of UTIs reduced the incidence and delayed the recurrence of UTIs [120]. The daily dosage of polyphenols was unknown. However, since 100 g of fresh cranberries contain 150–400 mg of polyphenols, depending on the cultivar, and the drying process reduces the weight of cranberries by about 4–5 times, it can be assumed that 42 g/day of dried cranberries could provide 63–168 mg of polyphenols per day [123].

#### 3.2.3. Nephro-/Urolithiasis

Two clinical trials investigated the effects of polyphenol extracts or polyphenol-containing beverages in patients with lithiasis. One trial was a cross-over study conducted in patients with CaOx kidney stones, who consumed two varieties of tea (i.e., Japanese green tea and South Africa herbal tea, both standardized in polyphenols) for 30 days (125 mL/day). The results showed that Japanese green tea reduced urine sodium, potassium, and brushite supersaturation, while South Africa herbal tea decreased urine volume. Crystal morphology shifted from mixed CaOx mono- and dihydrate to monohydrate after tea ingestion. No significant changes were observed in urinary and plasma oxidative stress biomarkers, such as TBARS [114]. The other study was a single-arm clinical trial that aimed to evaluate the potential effects of a commercially available pomegranate extract (POMx^TM^, standardized in polyphenols, from the whole fruit) in recurrent calcium-containing stone formers. Participants received POMx^TM^ capsules (1 g/day) for 3 months. Daily supplementation led to an increase in urinary levels of calcium, sodium, chloride, and magnesium, and showed a trend toward a reduction in CaOx supersaturation. In addition, POMx^TM^ increased the activity of serum paraoxonase 1 (PON1), an antioxidant enzyme that plays a central role in multiple pathological conditions, including kidney disease [124,125]. However, the increase in PON1 activity was not accompanied by a reduction in urinary and plasma biomarkers of oxidative stress and inflammation, such as TBARS, urinary 8-hydroxy-deoxyguanosine (8-OHdG), and C-reactive protein (CRP) [115].

#### 3.2.4. Other Genitourinary Pathological Conditions

A double-blind, placebo-controlled RCT investigated the effects of Equopausa^®^, a commercial product standardized in polyphenols and composed of fermented soy (from *Glycine max* L. seeds) and resveratrol, in menopausal women with vaginal dryness and bladder symptoms. Participants received Equopausa^®^ tablets (200 mg/day of fermented soy and 25 mg/day of resveratrol) for 3 months. Daily supplementation led to a marked decrease in the number of women experiencing mild bladder symptoms and moderate vaginal dryness compared to the placebo group (13.3% vs. 63.3% and 13.3% vs. 89.9%, respectively) [110].

Another study focused on patients with diabetes-associated albuminuria. It was a double-blind, placebo-controlled RCT in which participants received EGCG, epigallocatechin, and/or epicatechin (~800 mg of EGCG/day, orally) for 3 months. At the end of the follow-up period, green tea polyphenols reduced both micro- and macroalbuminuria and the urinary albumin-to-creatinine ratio compared to placebo. A significant percentage of patients experienced an improvement in their albuminuria status (19.0% in the intervention group vs. 0.0% in the control group). Finally, green tea polyphenols reduced circulating levels of the pro-inflammatory marker TNF-α but had no effect on blood CRP or urinary 8-isoprostane, a marker of oxidative stress [117].

Regarding BPH, only one double-bind, placebo-controlled RCT examined the effects of polyphenol extracts in this patient population. A 2-month oral supplementation with whole tomato extract standardized in polyphenols (5 g/day) reduced the International Prostate Symptom Score (IPSS), as well as urination frequency and urgency. In addition, it improved quality of life and showed a trend toward a reduction in PSA levels, particularly in patients with basal levels greater than 10 ng/mL. However, no significant changes were observed in free PSA concentrations or the free/total PSA ratio [109].

One placebo-controlled RCT (with unknown masking) was conducted in patients with chronic inflammatory prostatitis and multiple signs and symptoms of prostatic fibrosis. Participants received resveratrol tablets (dose unknown) daily for 2 months. The results showed a decrease in both the National Institutes of Health—Chronic Prostatitis Symptom Index (NIH-CPSI) score and the total IPSS compared to placebo. Moreover, resveratrol reduced the number of patients with severe symptoms (IPSS > 20), increased the expressed prostate secretion (EPS) volume after prostate massage, and decreased the number of white blood cells in EPS or post-massage urine [119].

#### 3.2.5. None

Three studies focused on individuals without genitourinary diseases but investigated the effects of isolated polyphenols or polyphenol extracts on genitourinary outcomes. One was a double-blind, placebo-controlled RCT, which demonstrated that a 4-month supplementation with trans-resveratrol tablets (75 or 500 mg/day) in men with metabolic syndrome reduced concentrations of androstenedione, dehydroepiandrosterone, and dehydroepiandrosterone-sulphate at the highest dose, suggesting a potential effect in preventing BPH. However, trans-resveratrol did not alter the levels of sex steroid hormones, prostate volume, or circulating PSA levels [118]. In a double-blind, randomized, cross-over trial, acute supplementation (3 h) with a cranberry leaf extract beverage (15.2 oz) or low-calorie cranberry juice (16.0 oz) in healthy adults promoted the anti-adhesive activity of urinary bacteria compared to placebo, indicating potential benefits for the prevention of UTIs, but had no effect on urinary creatinine excretion. In addition, low-calorie cranberry juice increased circulating GSH levels and serum SOD activity, and reduced IL-4 levels. However, no changes were observed in other antioxidant or anti-inflammatory markers following acute administration [107]. Finally, a cross-over study (with masking unknown) demonstrated that oral supplementation with AZO^®^ cranberry extract (1600 mg/day, standardized in polyphenols, from the whole fruit) or Ellura^®^ cranberry juice extract (265 mg/day, standardized in polyphenols) in healthy volunteers was effective in reducing urinary bacterial adhesion. Notably, the juice extract product (Ellura^®^) showed significantly higher activity compared to the whole berry product (AZO^®^) [106].

#### 3.2.6. Summary

The results of clinical trials conducted on isolated polyphenols or polyphenol-rich extracts, products, or foods (Table 1 and Table S2) have partially confirmed those of preclinical studies. For example, consumption of Japanese green tea, standardized for polyphenols, led to a change in crystal morphology from mixed forms of CaOx mono- and dihydrate to monohydrate forms in patients with nephrolithiasis, while a commercially available pomegranate extract (POMx^TM^) reduced CaOx supersaturation in individuals with recurrent calcium-containing stones. However, unlike what was observed with isolated polyphenols in rodent models, neither green tea nor POMx™ had significant effects on markers of oxidative stress.

Regarding prostate disorders, supplementation with tomato extract improved quality of life and reduced PSA levels in patients with BPH, while resveratrol was effective in reducing symptom severity in patients with chronic inflammatory prostatitis.

Promising results have also been observed in women with vaginal dysbiosis or vaginosis, where (i) cranberry juice consumption restored the vaginal microbiota; (ii) *Cymbopogon olivieri* extract ameliorated signs and symptoms of bacterial vaginosis, with effects superimposable to those of the reference compound metronidazole; (iii) wax propolis reduced the growth of *Candida albicans*; (iv) Micotirosolo^®^ (a commercial product particularly rich in hydroxytyrosol) lowered clinical symptoms and improved quality of life; and (v) Equopausa^®^, a commercial product standardized in polyphenols and composed of fermented soy, from *Glycine max* L. seeds, and resveratrol, reduced vaginal dryness and bladder symptoms in menopausal women.

A final potential application of polyphenol-rich extracts or products lies in the prevention and treatment of recurrent UTIs in both children and adults. A mixture of extracts of *Castanea sativa* Mill., *Serenoa repens* (W. Bartram) Small, and *Vaccinium macrocarpon* Ait. prevented the recurrence of UTIs, while supplementation with a cranberry extract syrup resulted in a UTI rate comparable to that observed in the trimethoprim-treated control group. In addition, daily consumption of sweetened dried cranberries has been found to reduce the incidence and delay the recurrence of UTIs (Table 1 and Table S2).

## 4. Conclusions

This systematic review paves the way for new potential uses of isolated polyphenols or polyphenol-rich extracts/products in the management of common genitourinary diseases, with a particular focus on the treatment of bacterial vaginosis and vaginal dysbiosis in women, as well as on the prevention of the recurrence of UTIs in both adults and children.

This work has several limitations. First, many of the extracts or products tested in the included studies were not standardized for polyphenol content, and some clinical trials were open-label or single-arm studies, introducing a potential risk of bias that should not be underestimated when interpreting the results. In addition, the risk of bias of the primary studies was not assessed, and a statistical data synthesis was not performed due to the high heterogeneity of animal models, patient populations, and outcomes. Future studies focusing on isolated compounds or standardized formulations and employing robust study designs are needed to confirm the preliminary evidence on polyphenols and to clarify the biological effects of ITCs and their natural sources in this field.

## Figures and Tables

**Table 1:** Summary of the main findings of the preclinical and clinical studies on isolated polyphenols, polyphenol-rich extracts, products, or foods included in the systematic review. Further details are provided in Tables S1 and S2. Symbols: *, gallic acid/ellagic acid/protocatechuic acid/pyrogallic acid; ^#^ standardized in polyphenols; ↓ decreased; ↑ improved.

Polyphenol/ Extract/Product/Food	N. of Studies	Main Findings
Preclinical studies (rodent models of genitourinary diseases)		
Resveratrol	9	↓ Kidney injury in nephrolithiasis; ↓ hyperoxaluria; ↓ histological alterations in prostatitis; ↓ inflammation, oxidative stress, and prostate weight in BPH; ↓ glomerulonephritis
EGCG	4	↓ Kidney injury in nephrolithiasis; ↓ inflammation, oxidative stress, and prostate weight in BPH; ↓ glomerulonephritis
Quercetin	2	↓ Kidney injury in nephrolithiasis
Protocatechuic acid	1	↓ Inflammation, oxidative stress, and prostate weight in BPH
Rutin	1	↓ CaOx levels in urolithiasis
Curcumin	1	↓ CaOx levels in urolithiasis
Epicatechin	1	↓ Renal CaOx deposition and renal injury score in nephrolithiasis
Piperine	1	↓ Glomerulonephritis
Oligonol	1	↓ Prostatic histological alterations in prostatitis
Rosmarinic acid	1	↓ Oxidative stress, inflammation, bladder weight, and/or urothelial thickness in cystitis
Combinations of citric acid + polyphenols *	1	↓ Renal CaOx deposition and renal injury score in nephrolithiasis
Unspecified green tea polyphenol	1	↓ Hyperoxaluria
Diosmin	1	↓ Inflammation, oxidative stress, and prostate weight in BPH
Caffeic acid	1	↓ Kidney injury in nephrolithiasis
Pterostilbene	1	↓ Oxidative stress, inflammation, bladder weight, and/or urothelial thickness in cystitis
Clinical studies		
Resveratrol	2	↓ Symptom severity in patients with chronic inflammatory prostatitis and multiple symptoms and signs of prostatic fibrosis; no effect on prostate volume in men with metabolic syndrome
Cranberry extract ^#^	2	↓ UTIs recurrence in children with a history of recurrent UTIs; ↑ urinary bacterial anti-adhesion activity in healthy adults
Cranberry juice ^#^	2	Restored the vaginal microbiota in postmenopausal women with vaginal dysbiosis; ↑ urinary bacterial anti-adhesion activity in healthy adults
Sweetened dried cranberries	1	↓ UTIs incidence and recurrence in women with a history of UTIs
Green tea polyphenols	1	Improved micro-/macroalbuminuria status and urinary albumin-to-creatinine ratio in patients with diabetes
Japanese green tea ^#^	1	↑ CaOx monohydrate forms (from mixed mono- and dihydrate forms) in patients with nephrolithiasis
Pomegranate extract ^#^	1	↓ CaOx supersaturation in individuals with recurrent calcium-containing stones
Whole tomato extract ^#^	1	↑ Quality of life and ↓ PSA levels in patients with BPH
*Cymbopogon olivieri* extract ^#^	1	↓ Signs and symptoms of bacterial vaginosis
Wax propolis	1	*↓ Candida albicans* growth in women with vaginal candidiasis
Hydroxytyrosol, Tea Tree Oil, *Tabebuia avellanedae* cortex extract, and *Juglans regia* L. leaf extract ^#^	1	↓ Clinical symptoms and ↑ quality of life in women with recurrent vulvovaginal candidiasis
Combination of resveratrol and fermented soy from *Glycine max L.* seeds ^#^	1	↓ Vaginal dryness and bladder symptoms in menopausal women
*Castanea sativa* Mill., *Serenoa repens* (W. Bartram) Small, and *Vaccinium macrocarpon* Ait. extracts ^#^	1	↓ UTIs recurrence in nephropathic patients with recurrent UTIs

## Data Availability

No new data were created or analyzed in this study. Data sharing is not applicable to this article.

## Supplementary Materials

The following supporting information can be downloaded at: https://www.mdpi.com/article/10.3390/ijms27041660/s1.

## Abbreviations

The following abbreviations are used in this manuscript:

BPH	Benign prostatic hyperplasia
CaOx	Calcium oxalate
CAT	Catalase
COX-2	Cycloxigenase-2
CRP	C-reactive protein
EGCG	Epigallocatechine-3-gallate
EPS	Expressed prostate secretion
GCLC	Glutamate-cysteine ligase catalytic subunit
GCLM	Glutamate-cysteine ligase modifier subunit
GLs	Glucosinolates
GPx	Glutathione peroxidase
GR	Glutathione reductase
GSH	Glutathione
GST	Glutathione-S-transferase
HO-1	Heme oxygenase-1
IL	Interleukin
iNOS	Inducible nitric oxide synthase
IPSS	International Prostate Symptom Score
ITCs	Isothiocyanates
LPO	Lipid peroxidation
MDA	Malondialdehyde
MPO	Myeloperoxidase
NF-κB	Nuclear factor kappa-light-chain-enhancer of activated B cells
NIH-CPSI	National Institutes of Health-Chronic Prostatitis Symptom Index
NLRP3	NOD-like receptor family, pyrin domain containing 3
NO	Nitric oxide
NQO1	NAD(P)H:quinone oxidoreductase 1
Nrf2	Nuclear factor erythroid 2-related factor 2
PON1	Paraoxonase 1
PSA	Prostate-specific antigen
RCT	Randomized controlled trial
ROS	Reactive oxygen species
SIRT1	Sirtuin 1
SOD	Superoxide dismutase
TBARS	Thiobarbituric acid reactive substances
TGF-β	Transforming growth factor-β
TNF-α	Tumor necrosis factor-α
UTIs	Urinary tract infections
VEGF	Vascular endothelial growth factor
XO	Xanthine oxidase
