# Supplementary material for: Potential Efficacy of Polyphenols and Isothiocyanates in the Management of Genitourinary Diseases: A Systematic Review of Preclinical and Clinical Studies
Source: Int J Mol Sci. 2026 Feb 8;27(4):1660. doi: 10.3390/ijms27041660 (PMC12940174; doi:10.3390/ijms27041660)
Supplement: Supplementary file 1 [file ijms-27-01660-s001.zip › ijms-4115439-supplementary.pdf]

S1. Search strategy.

Medline:

(polyphenols OR isothiocyanates OR hydroxytyrosol OR sulforaphane) AND (genitourinary OR genitourinary condition* OR genitourinary tract OR urinary tract infection* OR UTIs OR uropathy* OR nephrolithiasis OR prostatitis OR urethritis OR urinary incontinence OR benign prostatic hyperplasia OR BPH OR cystitis OR kidney stones OR nephritis OR lower urinary tract symptoms OR vulvovaginitis OR bacterial vaginosis OR vaginal infection)

Scopus:

TITLE-ABS-KEY (polyphenols OR isothiocyanates OR hydroxytyrosol OR sulforaphane) AND (genitourinary OR (genitourinary AND condition*) OR (genitourinary AND tract) OR (urinary AND tract AND infection*) OR UTIs OR uropathy* OR nephrolithiasis OR prostatitis OR urethritis OR (urinary AND incontinence) OR (benign AND prostatic AND hyperplasia) OR BPH OR cystitis OR (kidney AND stones) OR nephritis OR (lower AND urinary AND tract AND symptoms) OR vulvovaginitis OR (bacterial AND vaginosis) OR (vaginal AND infection))

Table S1. Main findings of the pre-clinical studies included in the systematic review. Abbreviations: bFGF, basic fibroblast growth factor; BPH, benign prostatic hyperplasia; CaOx, calcium oxalate; CAT, catalase; COX-2, cyclooxygenase-2; EGCG, epigallocatechin-3-gallate; EGF, endothelial growth factor; GCLC, glutamate-cysteine ligase catalytic subunit; GCLM, glutamate-cysteine ligase modifier subunit; GPx, glutathione peroxidase; GR, glutathione reductase; GSH, glutathione; GST, glutathione-S-transferase; HIF-1α, hypoxia-inducible factor 1α; HO-1, heme oxygenase-1; IL, interleukin; iNOS; inducible nitric oxide synthase; ITC, isothiocyanate; LPO, lipid peroxidation; MDA, malondialdehyde; MPO, myeloperoxidase; NLRP3, NOD-like receptor family, pyrin domain containing 3; NF-κB, nuclear factor kappa-light-chain-enhancer of activated B cells; NO, nitric oxide; NQO1, NAD(P)H:quinone oxidoreductase 1; Nrf2, nuclear factor erythroid 2-related factor 2; PGE2, prostaglandin E2; PSA, prostate-specific antigen; ROS, reactive oxygen species; SIRT1, sirtuin 1; SOD, superoxide dismutase; TBARS, thiobarbituric acid reactive substances; TGF-β, transforming growth factor-β; TNF-α, tumor necrosis factor-α; VEGF, vascular endothelial growth factor; XO-1, xanthine oxidase. Letters and symbols: * without statistical analysis; ↓ decrease; ↑ increase; [a] co-treatment from day 1; [b] co-treatment after 10 weeks of high-fat diet; [c] following established chronic prostatitis; [d] after 3 weeks of treatment with cationic bovine serum albumin; [e] after surgery; [f] after 15 days of treatment with sodium oxalate; [g] pre-treatment for 2 days; [h] on day 7 following anti-glomerular basement membrane antibody administration; [i] at the end of treatment with 17-β-estradiol; [l] 16 days after the end of treatment with prostatein; [m] pre-treatment for 18 days; [n] from the 1st day after procedure; [o] co-treatment from the 4th week; [p] co-treatment from day 2.

First author, year	*Rodent model*	*Compound*	*Daily dosage, administration route*	*N. of animals (control/ treated)*	*Lenght of treatment*	*Main results (efficacy)*	*Histological analysis**	*Main results (inefficacy, side effects, or no statistical analysis performed)*	*Circulating markers*
Akanni, 2020	Testosterone propionate-induced BPH in castrated male Wistar rats	Protocatechuic acid	40 mg/kg, *per os* (by gavage)	7/7	28 days[a]	↓ Prostate weight, MDA levels, LPO, MPO activity and NO levels; ↑ SOD activity.	↓ Prostatic hyperplasia but not histological changes.	Protocatechuic acid did not reduce the activity of prostatic GST.	↓ MPO activity, and NO, IL-1β and TNF-α levels.
Beklemisheva, 2007	Testosterone propionate-induced BPH in male Wistar rats	Phenetyl ITC	12 µmol/rat, *per os* (by gavage)	5/7	3 weeks[a]	↓ Prostate weight and hyperplasia.	Moderate ↓ in micropapillary structures; persistent ↑ nucleus-to-cytoplasm ratio. Vesicular nuclei, prominent nucleoli, and vacuolated cytoplasm observed.	↓ Androgen receptor expression and testosterone-mediated cell cycle progression (no statistical analysis performed).	-
Calmasini, 2018	Obesity-induced BPH in male C57BL6/J mice	Resveratrol	100 mg/kg, *per os* (by gavage)	Unknown	2 weeks[b]	↓ Prostate weight, hyperplasia, prostate contractility *ex vivo*, and ROS production.	-	-	-
Ghodasara, 2010	Ammonium chloride + ethylene glycol-induced CaOx urolithiasis in male Wistar rats	Rutin and curcumin	Rutin 20 mg/kg, curcumin 60 mg/kg, *per os* (drinking water)	6/6	28 days[a]	↓ Urinary and renal calcium and oxalate levels, as well as LPO and renal CaOx deposits.	↓ Kidney damage, interstitial fibrosis, and eosinophilic infiltration.	Curcumin, but not rutin, ↓ urine volume.	↓ Serum creatinine levels.
Grases, 2015	Ammonium chloride + ethylene glycol-induced nephrolithiasis in male Wistar rats	Epicatechin	200 mg/l, *per os* (drinking water)	8/8	24 days[a]	↓ Renal calcium content; ↑ urinary creatinine; ↓ urinary calcium, phosphorus, and pH.	-	No significant effects observed on diuresis, crystal deposition, urinary oxalate and magnesium levels, or renal phosphorus and magnesium levels.	-
Guzel, 2021	Ethylene glycol-induced nephrolithiasis in male Wistar rats	Quercetin	10 mg/kg, *per os* (by gavage)	8/8	5 weeks[a]	↓ Urinary oxalate, plasma urea, and plasma oxalate levels.	Mild ↓ in glomerular vessel congestion, mononuclear cell infiltration, and tubular lumen narrowing, particularly in the proximal tubules.	No statistically significant difference in urinary calcium levels.	↓ MDA levels.
He, 2017	Prostatic protetin-induced autoimmune chronic prostatitis in male Sprague-Dawley rats	Resveratrol	10 mg/kg, *per os*	8/8	10 days[c]	↓ Inflammatory cells infiltration and collagen accumulation. Improvement in overactive bladder: ↓maximum capacity, residual urine volume and maximum voiding pressure.	↓ Prostatic epithelial height and organization of the bladder detrusor muscle layer.	-	-

Hong, 2013	Ammonium chloride + ethylene glycol-induced hyperoxaluria in Sprague-Dawley rats	Resveratrol	5 and 10 mg/kg, *per os* (by gavage)	6/6	21 days[a]	↓ Number of urinary CaOx crystals.	-	Dose-dependent ↑ in the renal expression of GPx, CAT, and SOD (no statistical analysis performed).	↓ MDA levels.
Kakalij, 2016	Cationic bovine serum albumin-induced immune glomerulonephritis in BALB/c mice	Resveratrol or piperine	40 mg/kg, *per os*	6/6	3 weeks[d]	↓ Albuminuria and renal TBARS; ↑ urinary uric acid, renal GSH levels and SOD activity. Piperine: ↑ renal CAT activity.	-	-	↓ Serum uric acid and creatinine levels, and blood urea nitrogen.
Kim, 2009	Beta-estradiol and dihydrotestosterone-induced prostatitis in male Wistar rats	Oligonol	60 mg/kg, *per os* (drinking water)	10/10	4 weeks[a]	↑ GPx activity.	↓ Inflammatory cells and prostate inflammation severity; improved gland and stromal epithelium.	Oligonol did not restore prostate weight nor reduce plasma TNF-α levels but showed a tendency to enhance tissue SOD activity.	↑ Plasma GPx activity.
Kitamura, 2023	HCl-induced cystitis in female Sprague-Dawley rats	Rosmarinic acid	50 mg/kg, *per os*	7/7	3 days[e]	↑ Micturition interval; ↓ PGE2 release, urothelial thickness, inflammatory markers (IL-6), and MPO activity.	-	No significant change in micturition volume and bladder weight.	-
Li, 2023a	Glyoxylate-induced oxalate nephrolithiasis in male C57BL/6 mice	Combinations of citric acid + gallic acid/ellagic acid/ protocatechuic acid/pyrogallic acid	50 mg/kg, i.p.	5/5	6 days[a]	-	↓ Renal CaOx deposition and renal injury score.	↓ Blood urea and serum creatinine levels (no statistical analysis performed).	-
Li, 2023b	Sodium oxalate-induced nephrolithiasis in male albino rats	EGCG, quercetin or both (1:1)	EGCG: 150 mg/kg, quercetin: 10 mg/kg, *per os* (by gavage)	6/6	13 days[f]	↓ Urinary urea, creatinine, calcium, uric acid, phosphorus, potassium, and chloride; ↓ renal CAT activity and LPO.	↓ Crystals and dilated renal tubules.	-	↓ Serum urea, creatinine, calcium, uric acid, phosphorus, potassium, and chloride.
Li, 2021	Ammonium chloride + ethylene glycol-induced hyperoxaluria in Sprague-Dawley rats	Green tea polyphenol	10, 30 and 300 mg/kg, *per os* (by gavage)	10/10	4 weeks[a]	↓ Protein expression of Nrf2, HO-1, and NQO1 in the kidney (highest dose).	Dose-dependent ↓ in kidney swelling and stone formation. The highest dose restored kidney shape, tissue structure, and normal glomeruli.	-	-
Lu, 2020	Obesity-induced glomerulonephritis in C57BL/6J mice	Sulforaphane	1 mg/kg, i.p.	10/10	16 weeks[a]	↓ Urinary albumin-to-creatinine ratio, glomerular volume, and kidney damage.	-	-	-
Oksay, 2017	Ethylene glycol-induced nephrolithiasis in male Wistar rats	Resveratrol	10 mg/kg, *per os* (by gavage)	8/8	5 weeks[a]	↓ Urinary/plasma oxalate levels and plasma urea levels.	↓ Histopathological changes: glomerular vessel congestion (renal medulla and cortex), mononuclear cell infiltration, and narrowing of tubular lumen, mainly in proximal tubules.	No statistically significant difference in serum and urinary calcium levels.	↓ MDA levels.
Peng, 2011_ part I	Immune-mediated glomerulonephritis in male 129/SvJ mice	EGCG	50 mg/kg, *per os*	14/14	15 days[g]	↓ Proteinuria, histopathological changes (glomerulonephritis score, crescents, tubulointerstitial injury score), macrophage and lymphocyte infiltration, MDA and H_2O_2 levels (renal tissue and urine), renal MPO and iNOS expression, and total NO metabolites (kidney and urine); ↑ renal GPx expression and CAT activity.	-	No effects on nitrosative stress in the kidney tissue.	↓ Total NO metabolite and serum creatinine levels.
Peng, 2011_ part II	Immune-mediated glomerulonephritis in male 129/SvJ mice	EGCG	50 mg/kg, *per os*	14/14	21 days[h]	↓ Proteinuria and mortality rate.	-	-	↓ Serum creatinine levels.
Qian, 2021	17-β-estradiol-induced chronic prostatitis in Sprague-Dawley male rats	Resveratrol	10 mg/kg, *per os* (by gavage)	8/8	10 days[i]	↓ Prostatic levels of pro-inflammatory markers (IL-6, IL-18, and TNF-α).	↓ Interstitial inflammatory cell infiltration and fibroblastic hyperplasia; ↑ glandular secretions.	-	-

Song, 2023	Prostatein-induced chronic nonbacterial prostatitis in Sprague-Dawley male rats	Resveratrol	20 mg/kg, *per os* (by gavage)	7/7	10 days[l]	↓ Inflammatory infiltration, fibrosis and pro-inflammatory markers in prostatic tissue (IL-1β, TNF-α); ↑ SIRT1 and ↓ NF-kB expression.	-	-	-
Vafa, 2020	Propionate-induced BPH in male Wistar rats	Diosmin	20 and 40 mg/kg, *per os*	6/6	28 days[m]	Dose-dependent ↓ LPO and XO activity; ↓ MDA and uric acid levels; ↑ CAT, GST, GPx, GR, and GSH levels in prostatic tissue.	↓ Histopathological alterations (epithelial thickening, ↑ papillary fronds, and ↓ lumen area).	-	↓ Serum PSA levels.
Wan, 2017	Stress urinary incontinence due to vaginal distention in Sprague-Dawley female rats	Sulforaphane	0.5 mg/kg, i.p.	6/6	30 days[n]	↑ Peak voiding pressure and micturition interval; ↓ MDA levels; ↑ GPx and SOD activity in the urethra; ↓ cell apoptosis in the urethral sphincter.	Normalization of urethral sphincter muscle histology and collagen content.	-	↓ MDA levels.
Yasir, 2018_part I	Glycol-induced nephrolithiasis in male Wistar rats	Caffeic acid	20 and 40 mg/kg, *per os* (by gavage)	8/8	8 weeks[a]	↑ Urinary volume (40 mg/kg only) and urinary citrate excretion (both); ↓ urinary oxalate excretion (both).	Both dosages restored normal renal architecture and cleared crystal deposits from renal tubules.	-	-
Yasir, 2018_part II	Glycol-induced nephrolithiasis in male Wistar rats	Caffeic acid	20 and 40 mg/kg, *per os* (by gavage)	8/8	8 weeks[o]	↑ Urinary volume (40 mg/kg only) and urinary citrate excretion (both); ↓ urinary oxalate excretion (both).	Both dosages restored normal renal architecture and cleared crystal deposits from renal tubules.	-	-
Ye, 2015	Immune-mediated glomerulonephritis in male 129/SvJ mice	ECGC	25 and 50 mg/kg, *per os* (by gavage)	22/18	3 weeks[h]	↓ Proteinuria, mortality rate, and macrophages/lymphocytes infiltration in kidneys; amelioration of renal injury (glomerular injury, crescent formation, tubulointerstitial injury); ↑ renal GSH levels; ↓ renal and urinary MDA levels; ↑ Nrf2, GCLC, GCLM, SIRT1 and GPx expression in the kidney.	-	No significant effect on the expression of HO-1 in the kidney.	↓ Serum creatinine levels.
Yu, 2017	Prostatic protetin-induced autoimmune chronic prostatitis in Sprague-Dawley male rats	Resveratrol	10 mg/kg, *per os*	6/6	10 days[c]	↓ Bladder maximum capacity, residual urine volume and maximum voiding pressure.	-	-	-
Zeng, 2018	Prostatic protetin-induced autoimmune chronic prostatitis in Sprague-Dawley male rats	Resveratrol	10 mg/kg, *per os*	8/8	10 days[c]	↓ Bladder maximum capacity, residual urine volume and maximum voiding pressure; ↓ mast cell counts and fibrosis markers in prostatic tissue (TGF-β).	↓ Severe diffuse inflammation and collagen accumulation in the kidney; restoration of gland lumen diameter.	-	-
Zhang, 2025	Cyclophosphamide-induced interstitial cystitis in Sprague-Dawley female rats	Pterostilbene	10, 20 and 40 mg/kg, *per os* (by gavage)	6/6	1 week[p]	Dose-dependent ↓ bladder bleeding, histopathological score, edema, pain, and inflammation (i.e., ↓ IL-1β, IL-18 and NLRP3 inflammasome); ↓ bladder weight and bladder weight/body weight ratio; ↓ MDA and caspase levels; ↑ HO-1, SOD, and Nrf2 levels in bladder tissue.	↓ Infiltrating inflammatory cells.	-	-
Zhou, 2018	Testosterone-induced BPH in male Wistar rats	EGCG	50 and 100 mg/kg, *per os* (by gavage)	6/6	4 weeks[a]	↓ Androgen and estrogen receptor expression, prostatic MDA levels, pro-inflammatory markers (IL-1β, IL-6, TNF-α), COX-2 activity, angiogenesis (i.e., ↓ VEGF, bFGF and EGF levels), markers of fibrosis (TGF-β and HIF-1α), and	↓ Prostatic morphology changes (epithelial cell expansion, papillary protuberance, collagen deposition).	-	-

						epithelial-mesenchymal transition in prostatic tissue; ↑ SOD, CAT, and GPx activity, as well as GSH and total thiols levels.			

Table S2. Main findings of the clinical studies included in the systematic review. Abbreviations: 8-OHdG, 8-hydroxy-deoxyguanosine; BPH, benign prostatic hyperplasia; CaOx, calcium oxalate; CRP, C-reactive protein; DHEA, dehydroepiandrosterone; DHEAS, dehydroepiandrosterone-sulphate; eGFR, estimated glomerular filtration rate; EGCG, epigallocatechin gallate; EPS, expressed prostate secretion; fl oz, fluid ounce; FORD, free oxygen radical defense; FORT, free oxygen radical test; GPx, glutathione peroxidase; GSH, glutathione; GSSG, oxidized glutathione; IL, interleukin; IPSS, International Prostate Symptom Score; LPO, lipid peroxidation; MetS, metabolic syndrome; NAG, N-acetyl-β-D-glucosaminidase; NIH-CPSI, National Institutes of Health – Chronic Prostatitis Symptom Index; NO, nitric oxide; PGE2, prostaglandin E2; PGI, patient impression of global improvement; PON1, paraoxonase 1; PSA, prostate-specific antigen; RCT, randomized controlled trial; SD, standard deviation; SOD, superoxide dismutase; T1D, type 1 diabetes; T2D, type 2 diabetes; TBARS, thiobarbituric acid reactive substances; TNF-α, tumor necrosis factor-α; UTI, urinary tract infection. Symbols: * standardized in polyphenols; ^ epigallocatechin gallate, epigallocatechin, and epicatechin; § including 80 mg of isoflavone aglycones and 10 mg of equol; ↓ decrease; ↑ increase.

First author, year	*Study design*	*Population*	*N. of patients (men, %) - intervention/ control*	*Mean age (years ± SD or range) - intervention/ control*	*Intervention/ control*	*Daily dosage, administration route (dosage form), follow-up*	*Main results*	*Circulating markers*
Al-Othaim, 2021	Double-blind, randomized, cross-over study	Postmenopausal women with or without vaginal dysbiosis	23/23 (0.0/0.0)	61.0 ± 7.0	Cranberry juice*/placebo	8 fl oz/day, *per os* (juice), 15 days	• Cranberry juice had minimal impact on the vaginal microbiota of women whose microbiota was dominated by *Lactobacillus*.<br>• In women with diverse baseline microbiota, cranberry juice reduced the number of taxa but maintained key *Firmicutes* (*Lactobacillus, Anaerococcus, Finegoldia,* and *Peptoniphilus*) and *Actinobacteria* (*Actinomyces, Varibaculum, Actinomycetales,* and *Corynebacterium*). In contrast, the placebo resulted in a significant reduction of these bacteria.<br>• In women with strong dysbiosis, characterized by a high initial abundance of *Streptococcus,* cranberry juice reduced the relative abundance of *Streptococcus* more effectively compared to placebo. Notably, *Streptococcus* was replaced by *Firmicutes* (*Anaerococcus* and *Finegoldia*) and *Actinobacteria* (*Actinobaculum, Actinomyces, Varibaculum*, and *Corynebacterium*).	-
Borges, 2016	Double-blind RCT	Patients with T1D or T2D and persistent micro- or macroalbuminuria	21/21 (47.8/66.7)	63 (60–65)/ 59 (49–63)	Green tea polyphenols^/ placebo	~ 800 mg of EGCG/day, *per os* (capsules), 3 months	• Micro-/macroalbuminuria status (% of patients) -> intervention: from 70% to 30%; placebo: from 42% to 58%.<br>• Urinary albumin-to-creatinine ratio (mg/g) -> intervention: from 210 (39–1267) to 133 (10–1812); placebo: from 427 (77–4051) to 452 (43–4802).<br>• Improvement in albuminuria stage: 19% of patients in the intervention group (macro/micro -> micro/normo); 0% in the placebo group.<br>• No significant change in urinary levels of 8-isoprostane.	↓ TNF-α but not CRP levels.
Burleigh, 2013	Single-arm clinical trial	Women with a history of UTIs	17/- (0.0/-)	37 (18-64)	Sweetened dried cranberries	42 g/day, 2 weeks	↓ Incidence of UTIs and delayed recurrence (9 of 17 patients reported no UTIs within six months of starting the study; mean UTI rate per 6 months: from 2.4 to 1.1).	-

Cormio, 2021	Double-blind RCT	Patients with BPH	20/20 (100.0/100.0)	65.6 ± 5.1/ 64.1 ± 8.2	Whole tomato extract*/placebo	5 g/day, *per os* (sachets), 2 months	• ↓ IPSS (from 9.05 ± 1.15 to 7.15 ± 1.04), as well as urination frequency and urgency.<br>• ↑ Quality of life.	Trend toward ↓ total serum PSA levels (from 8.98 ± 1.52 to 6.95 ± 0.75 ng/mL). No change in free PSA concentrations and free/total PSA ratio.
Davinelli, 2017	Double-blind RCT	Menopausal women with vaginal dryness and bladder symptoms	30/30 (0.0/0.0)	52.7 ± 2.1/ 52.1 ± 1.7	Fermented soy (from *Glycine max* L. seeds) + resveratrol (Equopausa®)*/ placebo	200 mg/day (fermented soy§) + 25 mg/day (resveratrol), *per os* (tablets), 3 months	• Women (%) with mild bladder symptoms at the end of treatment -> intervention: 13.3; placebo: 63.3.<br>• Women (%) with moderate vaginal dryness at the end of treatment -> intervention: 13.3; placebo: 89.9.	-
Esmaili, 2024	Double-blind RCT	Women with bacterial vaginosis	45/45 (100.0/100.0)	30.9 ± 7.6/ 32.6 ± 6.7	*Cymbopogon olivieri* extract (whole plant)*/ metronidazole	500 mg/day, vaginally (capsules), 1 week	Patients (%) with vaginal burning -> intervention: from 100.0 to 6.7; control: from 95.6 to 15.6; itching -> intervention: from 100.0 to 4.4; control: 82.2 to 13.3; malodor -> intervention: from 93.3 to 11.1; control: from 80.0 to 8.9; abundant vaginal discharge -> intervention: from 100.0 to 15.6; control: from 100.0 to 20.0; pH > 4.5 -> intervention: from 91.1 to 6.7; control: from 100.0 to 11.1; positive whiff test -> intervention: from 93.3 to 11.1; control: from 91.1 to 6.7; clue cells -> intervention: from 88.9 to 11.1; control: from 88.9 to 6.7.	-
Farida, 2020	RCT (masking unknown)	Women with vaginal candidiasis	20/20 (100.0/100.0)	20-50	Wax propolis (5.0%) extract/nystatin	One ovule/day, vaginally (ovules), 1 week	Wax propolis inhibited the growth of *C. albicans,* with no significant differences between groups. Dropouts due to infections -> intervention: 2; control: 3.	-
Howell, 2022	Cross-over study with a 5-day wash-out period (masking unknown)	Healthy subjects	20/20 (unknown)	25-60	AZO® cranberry extract (whole fruit)*/ Ellura® cranberry juice extract*	1600 mg/day (AZO®) or 265 mg/day (Ellura®), *per os* (capsules), 1 week	↑ Urinary bacterial anti-adhesion activity. The juice extract product (Ellura®) showed significantly higher activity compared to the whole berry product (AZO®).	-
Kjaer, 2015	Double-blind RCT	Men with MetS	21/21/24 (100.0/100.0/ 100.0)	48.6 ± 1.5/ 51.5 ± 1.3/ 47.3 ± 1.3	Trans-resveratrol/ placebo	75 or 500 mg twice daily, *per os* (tablets), 4 months	No significant effect on prostate volume.	High-dose trans-resveratrol: ↓ levels of androstenedione (24%), DHEA (41%), and DHEAS (50%). No change in other sex steroid hormones or PSA levels.
Mathison, 2014	Double-blind, randomized, cross-over study with a 7-day wash-out period	Healthy adults	12/12 (50.0/50.0)	27.5 ± 1.3	Cranberry leaf extract beverage (CLEB) or low-calorie cranberry juice (LCJC)*/ placebo	CLEB: 15.2 oz; LCJC: 16.0 oz; placebo: 15.2 oz; *per os* (beverage), 3 hours	• ↑ Urinary bacterial anti-adhesion activity.<br>• No change in urinary creatinine excretion.	LCJC: ↑ GSH levels and serum SOD activity, and ↓ IL-4 levels. CLEB: ↑ GPx activity. No change in GSSG, GSH:GSSG ratio, CRP levels, eicosanoids, ILs other than IL-4, NO, 8-OHdG, and total antioxidant capacity.
Noce, 2021	Controlled clinical trial (masking unknown)	Nephropathic patients with recurrent UTIs	16/10 (50.0/50.0)	65.5 ± 2.0/ 67 ± 1.9	*Castanea sativa* Mill., *Serenoa repens* (W. Bartram) Small, and *Vaccinium macrocarpon* Ait. extracts*/ no treatment	6.21 mg of polyphenols/day, *per os* (capsules), 6 weeks	• No change in renal function (creatinine and eGFR).<br>• ↓ Erythrocyte sedimentation rate in males (from 16.7 ± 2.2 mm/h to 11.3 ± 1.5 mm/h) but not in females.<br>• ↓ Leukocytes in urinary sediment -> males: from 43.5 (1–450) n/µl to 15 ± 5.7 n/µl; females: from 28.5 (1–990) n/µl to 7 (1–91) n/µl.<br>• ↓ Urinary bacterial flora in males: from 428 ± 143.4 n/µl to 34 (0–450) n/µl.<br>• UTI relapses -> intervention group: 0; control group: 3 (*Escherichia coli*: 2; *Enterococcus faecalis*: 1).	Males: ↓ FORT and ↑ FORD; females: ↑ FORT.

Rodgers, 2016	Cross-over study with a 7-day wash-out period (masking unknown)	Patients with CaOx kidney stones	8/8 (100.0/100.0)	30-60	Japanese green tea* (JGT) or South Africa herbal tea* (RT)	125 ml/day, *per os* (cups of tea), 30 days	• RT group: ↓ urine volume.<br>• JGT group: ↓ urine sodium, potassium, and supersaturation of brushite.<br>• No change in other crystallization risk factors after ingestion of either tea.<br>• Crystal morphology shifted from mixed calcium oxalate mono- and dihydrate to predominantly monohydrate after tea ingestion.<br>• No change in urinary oxidative stress biomarkers (TBARS and NAG).	No change in plasma levels of TBARS.
Tracy, 2014	Single-arm clinical trial	Recurrent calcium-containing stone formers	23/- (43.0/-)	40.4/-	Pomegranate extract from the whole fruit (POMx™)*/-	1 g/day, *per os* (capsules), 3 months	• Trend toward ↓ supersaturation of CaOx.<br>• ↑ Urinary calcium, sodium, chloride, and magnesium.<br>• Trend toward ↑ urine volume.<br>• No change in urinary 8-OHdG levels.	↑ PON1 activity. No change in serum TBARS, LPO or CRP levels.
Uberos, 2015	Double-blind RCT	Children with a history of recurrent UTIs, with or without vesicoureteral reflux	76/116 (unknown)	1 month-13 years	Cranberry extract (Urell®)*/ trimethoprim	0.2 ml/kg/day (~ 5.6 mg/kg of cranberry extract/day), *per os* (syrup), maximum follow-up: 1 year	• Rate of UTIs in children < 1 year -> cranberry syrup: 35% (95% CI: 17–52); trimethoprim: 28% (95% CI: 13–42).<br>• Rate of UTIs in children > 1 year -> cranberry syrup: 26% (95% CI: 12–41); trimethoprim: 35% (95% CI: 21–50).<br>• Recurrent UTIs caused by *Escherichia coli* in 60% of cases (no difference between treatment groups).<br>• Multidrug-resistant bacteria in urine cultures -> cranberry syrup: 22.9%; trimethoprim: 33.3%.<br>• ↑ Urinary levels of hydroxycinnamic and hydroxybenzoic acids.	-
Vicari, 2020	RCT (masking unknown)	Patients with chronic inflammatory prostatitis and multiple symptoms and signs of prostatic fibrosis	32/32 (100.0/100.0)	35 (30-49)/ 35 (30-49)	Resveratrol/ placebo	Unknown, *per os* (tablets), 2 months	• Total NIH-CPSI score -> intervention: from 17.5 (14.0–22.0) to 14.0 (9.0–19.0); control: from 18.0 (14.0–23.0) to 19.5 (13.0–24.0).<br>• Total IPSS score -> intervention: from 19.5 (9.0–24.0) to 12.5 (7.0–22.0); control: from 19.0 (9.0–24.0) to 19.0 (12.0–24.0).<br>• Patients with severe symptoms (IPSS > 20) -> intervention: from 15.6% to 12.5%; control: from 18.7% to 21.8%.<br>• EPS volume (ml) after prostate massage -> intervention: from 0.05 (0.0–0.1) to 0.3 (0.0–0.5); control: no change.<br>• Patients without EPS after prostate massage -> intervention: from 87.5% to 40.6%: control: no change.<br>• White blood cells in expressed prostate secretion or post-massage urine -> intervention: from 11.0 (8.0–16.0) to 6.0 (5.0–9.0); control -> from 10.0 (8.0–15.0) to 12.0 (9.0–16.0).	-
Zullo, 2022	Single-arm clinical trial	Women with recurrent vulvovaginal candidiasis	60/- (0.0/-)	39.8 ± 13.5	Hydroxytyrosol, Tea Tree Oil, *Tabebuia avellanedae* cortex extract, and *Juglans regia* L. leaf extract (Micotirosolo®)*/-	Unknown, *per os* (tablets), 3 months	• ↓ Clinical symptoms and vaginal signs -> pruritus: from 5.0 ± 1.2 to 1.1 ± 0.8; burning: from 5.5 ± 1.4 to 1.3 ± 0.8; itching: from 3.9 ± 1.9 to 1.1 ± 0.8; vulvar erythema: from 4.1 ± 1.4 to 1.0 ± 0.5; vaginal discharge: from 5.0 ± 1.8 to 0.5 ± 0.6; tenesmus: from 3.2 ± 1.5 to 1.2 ± 0.7; vaginal dryness: from 4.6 ± 2.1 to 1.5 ± 0.9; dyspareunia: from 2.3 ± 1.2 to 0.7 ± 0.3.<br>• PGI-I after 3 months -> very much better: 51 patients (85%); much better: 3 patients (5%); a little better: 3 patients (5%); no improvement: 3 patients (5%); total success (very much better + much better): 54 patients (90%).	-
